# Transcriptome analysis reveals genes associated with wool fineness in merinos

**DOI:** 10.7717/peerj.15327

**Published:** 2023-05-23

**Authors:** Shengchao Ma, Li Long, Xixia Huang, Kechuan Tian, Yuezhen Tian, Cuiling Wu, Zhiwen Zhao

**Affiliations:** 1 College of Animal Science, Xinjiang Agricultural University, Urumqi, China; 2 Institute of Animal Science and Veterinary Medicine, Shandong Academy of Agricultural Sciences, Jinan, China; 3 Key Laboratory of Genetics Breeding and Reproduction of Xinjiang Wool Sheep and Cashmere-Goat, Institute of Animal Science, Xinjiang Academy of Animal Sciences, Urumqi, China

**Keywords:** Merinos, Wool fineness, RNA-Seq, DEGs

## Abstract

Hair/wool usually plays an important role in maintaining animal physiological activities, and the economic value of wool cannot be ignored. At present, people set higher demands on wool fineness. Hence, improving wool fineness is the concern of fine wool sheep breeding. Using RNA-Seq to screen the potential candidate genes that associate with wool fineness can provide theoretical references for fine-wool sheep breeding, and also provide us new ideas for further understand the molecular regulation mechanism of hair growth. In this study, we compared the expression pattern difference of genome-wide genes between the skin transcriptomes of Subo and Chinese Merinos. The results showed that, 16 candidate differentially expressed genes (DEGs) (Included: *CACNA1S*, *GP5*, *LOC101102392*, *HSF5*, *SLITRK2*, *LOC101104661*, *CREB3L4*, *COL1A1*, *PTPRR*, *SFRP4*, *LOC443220*, *COL6A6*, *COL6A5*, *LAMA1*, *LOC114115342* and *LOC101116863* genes) that may associate with wool fineness were screened, and these genes were located in signaling pathways that regulate hair follicle development, cycle or hair growth. It is worth noting that, among the 16 DEGs, *COL1A1* gene has the highest expression level in Merino skins, and the fold change of *LOC101116863* gene is the highest, and the structures of these two genes are both highly conserved in different species. In conclusion, we speculate that these two genes may play a key role in regulating wool fineness and respectively have similar and conserved functions in different species.

## Background

Hair/wool is a important epidermis appendage, tactile organ and a symbol of sexual dimorphism, which also plays an key role in animal physiological activities, for example, thermoregulation, protection from radiation, mosquito bites, and predator bites. In addition, the growth of hair/wool also reflects the health condition of animals and is affected when the body’s metabolism is disordered and malnutrition occurs (Rook, 1965; Berman, 1960; Farthing et al., 1982; Peters, Arck & Paus, 2006; Muneeb, Hardman & Paus, 2019; Pinter, 1968; Trüeb, 2015). Therefore, wool is an important material for studying the environmental suitability, social behaviors, and physiology of animals. The epidermis of fine wool sheep (*Ovis aries*) is completely covered by long and fine wool among mammals, wool is not only necessary to maintain its physiological activity, but also has important economic value. In modern society, wool is often regarded as an important textile raw material and widely used in textile industry. With the development of society, people have a higher demand for wool quality, wool textile products are developing towards high-end, thinner and softer. Wool fiber fineness directly determine the quality of wool textiles, it determines 75% of the value of the wool top, the variation of wool fiber diameter accounts for 61% of the total wool profit. In recent years, the output of sheep wool is increasing in China, but Chinese fine wool sheep mainly produce wool of 20–25 µm, and the output of wool below 18.5 µm is relatively lacking. Therefore, it is very necessary to continue to carry out the breeding of superfine wool sheep in the main producing areas of fine wool sheep.

Among the many phenotypic traits in livestock, wool fineness traits are quantitative trait, and their heritabilities are low to mid degree (Safari, Fogarty & Gilmour, 2005; Snyman et al., 1995; Hamadani et al., 2019). Hence, the accuracy of measurement of wool fineness traits is often affected by external factors, which makes it impossible to distinguish the contribution of genotype and environment to the phenotypic value of trait. This further resulted in the slow genetic progress of wool fineness. With the advancement of science and technology, understanding the genetic law or molecular regulation mechanism of wool traits, screening candidate genes or loci associated with wool fineness based on RNA-Seq, and applying them further to marker-assisted selection (MAS) and genome-wide selection (GS) to accelerate the genetic progress of sheep wool fineness traits are very innovative and foregrounded.

The hair/wool follicle is the control center of wool growth and development, and has a unique structure and the function of periodic regeneration. The wool phenotypes such as fiber fineness, fiber length, curvature, strength and elongation, and flexibility are directly affected by the development of hair/wool follicles (David et al., 2002; Purvis & Franklin, 2005). Then, the process of hair/wool follicle development is regulated by multiple genes and pathways. For example, in the study of Huelsken et al. (2001). High expression of Wnt/β-catenin signal promote the formation of the hair follicle placode and play a regulatory role in the process of hair follicle morphogenesis and redifferentiation. Demehri & Kopan (2009) found that the binding of Notch receptors to ligands will activate the hair follicle stem cells, so that the hair follicles enter the growth phase from the resting phase. Zhang et al. (2019) found that the MAPK and Hedgehog signaling pathways play an important regulatory role in the periodic cycle of hair follicles. In addition, the high expression of BMP signal prompts the hair follicle to enter the telogen phase, while the low expression of BMP signal prompts the hair follicle to enter the regeneration stage (Plikus et al., 2008). All of these known signaling pathways may be related to wool fineness, but the specific regulatory mechanisms remain to be further studied. In addition, a few scholars directly revealed more candidate genes related to wool fineness using DNA and RNA sequencing techniques, *e.g.*, Wang et al. (2014) and Zhao et al. (2021) found that *KIF16B* gene, *UBE2E3* and *RHPN2* genes associated with merino wool fiber diameter by using GWAS, respectively. Sulayman et al. (2018) found that three genotypes of *KRT36* gene were associated with Chinese merino wool fineness by using PCR-SSCP and DNA sequencing techniques. Shi et al. (2022) conducted transcriptome sequencing analysis on skin with different wool densities and found that *TNF*, *MAP2K2*, *INHBA*, *FST*, *PTPN11*, *MAP3K7*, *KIT* and *BMPR1A* genes may affect the wool density of Hetian sheep. However, previous reports are limited and cannot fully reveal the molecular regulation mechanism of wool fineness. In summary, we consider that it is still necessary to find new candidate regulatory genes.

Subo Merino are bred by crossbreeding, its male parent is Australian Merino and female parents are Xinji fine wool sheep, Chinese merino and Aohan fine wool sheep. Compared with their parents, Subo Merino have these basic features: high wool yield, good wool quality and fine wool (wool fiber diameter: 17–19 µm). It is a good material for studying the molecular regulation mechanism of wool fineness. In this study, we compared the genome-wide gene expression pattern between the skin tissues of Subo Merino (Superfine wool sheep, the wool spinning number >80 and fibre diameter from 17.0 to 18.5 µm of Superfine wool sheep) and Chinese Merino (Fine wool sheep, the wool spinning numbers and fibre diameter from 19.6 to 25 µm of fine wool sheep) by using RNA-Seq, and further screened the candidate genes associated with wool fineness. Functional enrichment and protein protein interaction network analysis were performed on DEGs to narrow the range of candidate genes. Finally, we conducted a series of bioinformatics analysis of some important candidate genes, such as protein domain prediction, phylogenetic analysis and selection pressure analysis. Overall, our results provide a theoretical basis for study the molecular regulatory mechanism of hair/wool growth and fineness and molecular breeding of Merinos.

## Methods and Materials

### Animals

**Figure 1:**
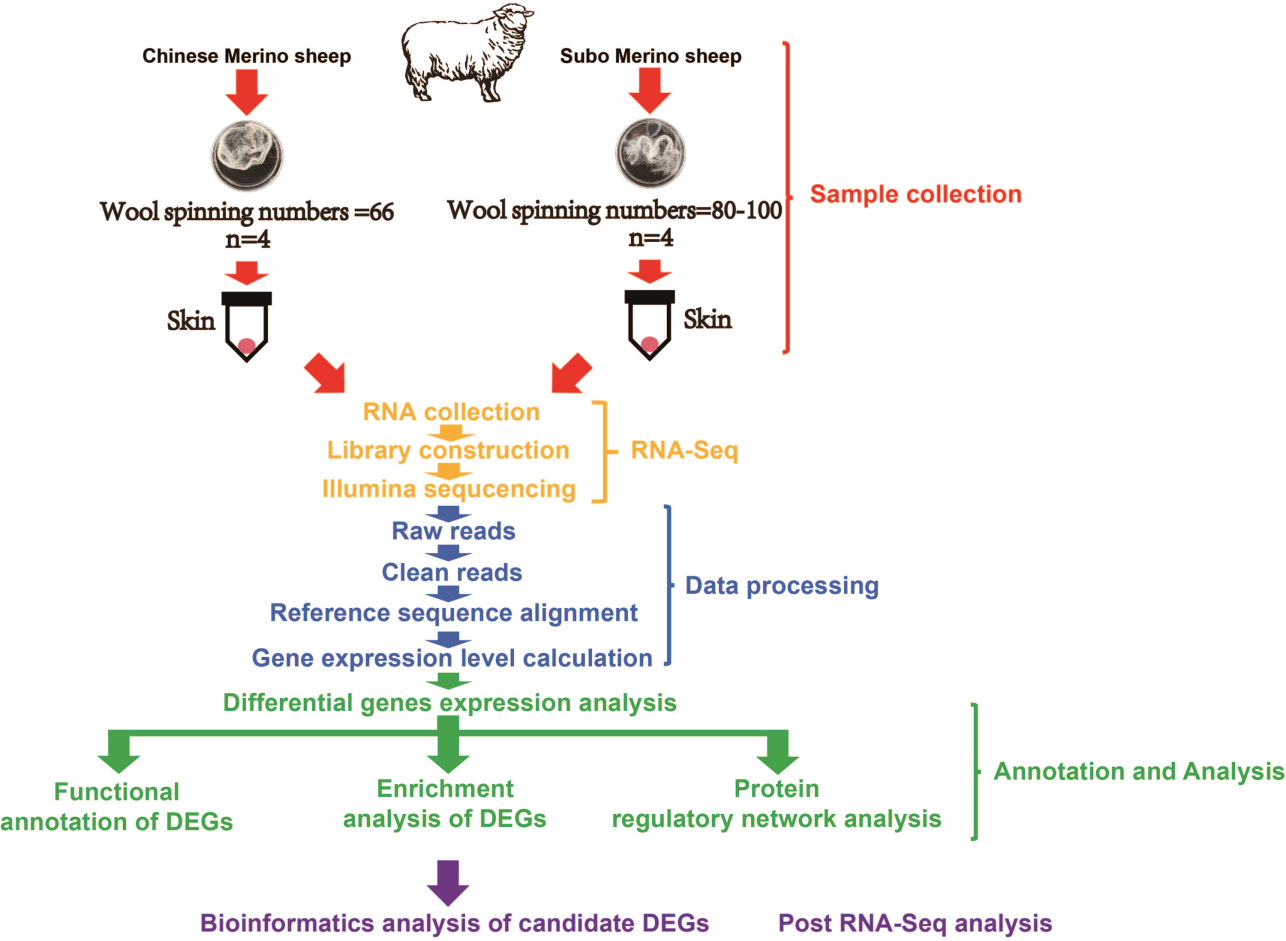
The design of experiments and technology road-map.

The experimental animals were provided by Gongnaisi Sheep Farm (Xinjiang, China). We selected 4 14-month-old Subo Merinos (Wool spinning numbers: 80–100; Superfine, SCe) and 4 14-month-old Chinese Merinos (Wool spinning numbers: 66; Fine, Ce) and took their skin tissues (skin tissue contained numerous hair follicles) (Fig. 1), put them into RNAlater for preservation, and finally transferred all samples to ultra-low temperature (−80 °C) freezer for long-term storage and used for RNA extraction. In addition, we provided a clean, warm and ventilated living environment for the experimental animals. During the breeding process, we carefully cared for these experimental animals to provided them with sufficient food and water. During the sampling process, we only took a small piece of skin tissue of the experimental animal and strictly disinfected and bandaged their wounds. The sampling process does not cause damage to the health of experimental animals, and their wounds soon heal.

### RNA extraction and purification

The total RNA extraction of eight skin tissue samples was completed by using TRIzol reagent (Invitrogen, Waltham, MA, USA), total RNA of eight samples was extracted based on the instruction of TRIzol reagent, and the quality control of eight total RNA samples was further performed. The results of quality control showed that the OD260/280 were all in the range of 1.8–2.0, the OD260/230 were all greater than 2.0, and the RIN values were all in the range of 7.0–8.5 of the eight total RNA samples, which could be used for subsequent experiments.

### Libraries construction and RNA-Seq

After the RNA samples were qualified, the Illumina-seq libraries of eight skin tissue samples was constructed, the reagent was provided by Illumina (San Diego, CA, USA), the eight libraries were constructed according to the instruction of Illumina. Finally, the qPCR was used to quantify the effective concentration of each library (library effective concentration > 2 nm). After the libraries were qualified, the eight libraries were pooled, and further sequenced by Illumina platform in Biomarker Technologies Co, LTD.

### Transcriptome data quality control and analysis

After RNA sequencing was finished, strict quality control of the raw data of eight samples was performed, and low-quality reads (low-quality reads: reads with a ratio of N greater than 10%; reads in which the bases of quality value Q ≤10 make up more than 50% of the entire reads) were removed. The clean data generated after quality control was used for subsequent analysis.

The transcriptomes of eight individuals were divided into two groups (Ce group and SCe group) based on their wool fineness phenotype, each containing four replicates. The statistical power of this experimental design, calculated in online implementation of RNASeqPower (https://rodrigo-arcoverde.shinyapps.io/rnaseq_power_calc/) is 0.79. The clean data were mapped to the sheep reference genome (ARS-UI_Ramb_v2.0, GCF_016772045.1) by using HISAT2 (Kim et al., 2019). Then, assembled and quantified the transcripts by using StringTie (Pertea et al., 2015), and fragments per kilobase million (FPKM) was regarded as a measure of transcript or gene expression level. DEG analysis was performed by using edgeR (Robinson, McCarthy & Smyth, 2010). During the identification of DEGs, the fold change was value calculated based on the FPKM of each gene, and the screening conditions were: Fold Change ≥1.5 and *P*-value < 0.05.

### Functional enrichment analysis and protein protein interaction network analysis of DEGs

GO (Gene Ontology) and KEGG (Kyoto Encyclopedia of Genes and Genomes) enrichment analysis of DEGs was performed by using the online tool DAVID (https://david.ncifcrf.gov/) with parameters set as default. The GO annotation is divided into three levels: biological process (BP), cellular component (CC) and molecular function (MF). Finally, the DEGs were submitted to the STRING database (https://cn.string-db.org/) to construct the protein protein interaction network. At the same time, Cytoscape software was used for visual editing of the interaction network (Smoot et al., 2011).

### Acquisition and analysis of gene nucleic acid and protein sequences

First of all, the COL1A1 and LOC101116863 (40S ribosomal protein S6-like) protein sequences of sheep were used for tblastn and blastp to obtain the mRNA and protein sequences of *COL1A1* and 40S ribosomal protein S6 genes of other seven species (Including: goat (*Capra hircus*), cattle (*Bos taurus*), human (*Homo sapiens*), rabbit (*Oryctolagus cuniculus*), dog (*Canis lupus familiaris*), mouse (*Mus musculus*) and chicken (*Gallus gallus*)). Nucleic acid and protein sequence alignments were then performed by using clustalx and clustalw (Larkin et al., 2007), respectively. Finally, the domains of the protein sequence were predicted by using the online tool MEME (https://meme-suite.org/meme/).

### Protein structure and phylogenetic analysis of genes

The mRNA sequences of eight species were submitted to MEGA 7.0 software to align (Kumar, Stecher & Tamura, 2016) and constructed a bootstrap tree (1,000 replicate) (Felsenstein, 1985) for *COL1A1* and *LOC101116863* genes. The maximum likelihood (ML) method was used for this analysis. Then, we checked the best DNA model for each gene (The best DNA model: *COL1A1*: GTR + G and *LOC101116863*: K2 + I). The ML search started with the initial tree generated by BioNJ (Gascuel, 1997), and the optimal tree was determined by using the NNI (nearest-neighbor interchange) algorithm.

### Synteny analysis

In synteny analysis, we checked the annotation of reference genomes from NCBI of 8 species, including reference genomes for sheep (ARS-UI_Ramb_v2.0, GCF_016772045.1), goat (ARS1.2, GCF_001704415.2), chicken (GRCg6a, GCF_000002315.6), cattle (ARS-UCD1.3, GCF_002263795.2), human (GRCh38.p14, GCF_000001405.40), rabbit (OryCun2.0, GCF_000003625.3), dog (ROS_Cfam_1.0, GCF_014441545.1) and mouse (GRCm39, GCF_000001635.27). These species are somewhat representative, and their reference genomes have been well annotated.

### Selection pressure analysis

In this study, the ratio of the non-synonymous substitution rate to the synonymous substitution rate (*ω* = dN/dS) of *COL1A1* and 40S ribosomal protein S6 genes was evaluated using the site model in the CODEML program in PAML4.9 software (Yang, 2007). The *ω* value is an important measure of selection pressure, where *ω* < 1, = 1, and > 1 mean purifying selection, neutral selection, and positive selection, respectively.

## Results

### Data quality control

After the eight libraries were sequenced, the generated raw data was further filtered to obtain the clean data. Finally, a total of 49.84 Gb clean data was obtained, the mean clean data size of each sample reached 6.07Gb, and the percentages of Q30 bases of eight samples were 92.25% and above. The clean data of each sample were mapped with the sheep reference genome, and the mapping rate ranged from 94.54% to 96.01% (Table S1).

### The expression of genes related to wool growth and follicle development

**Figure 2:**
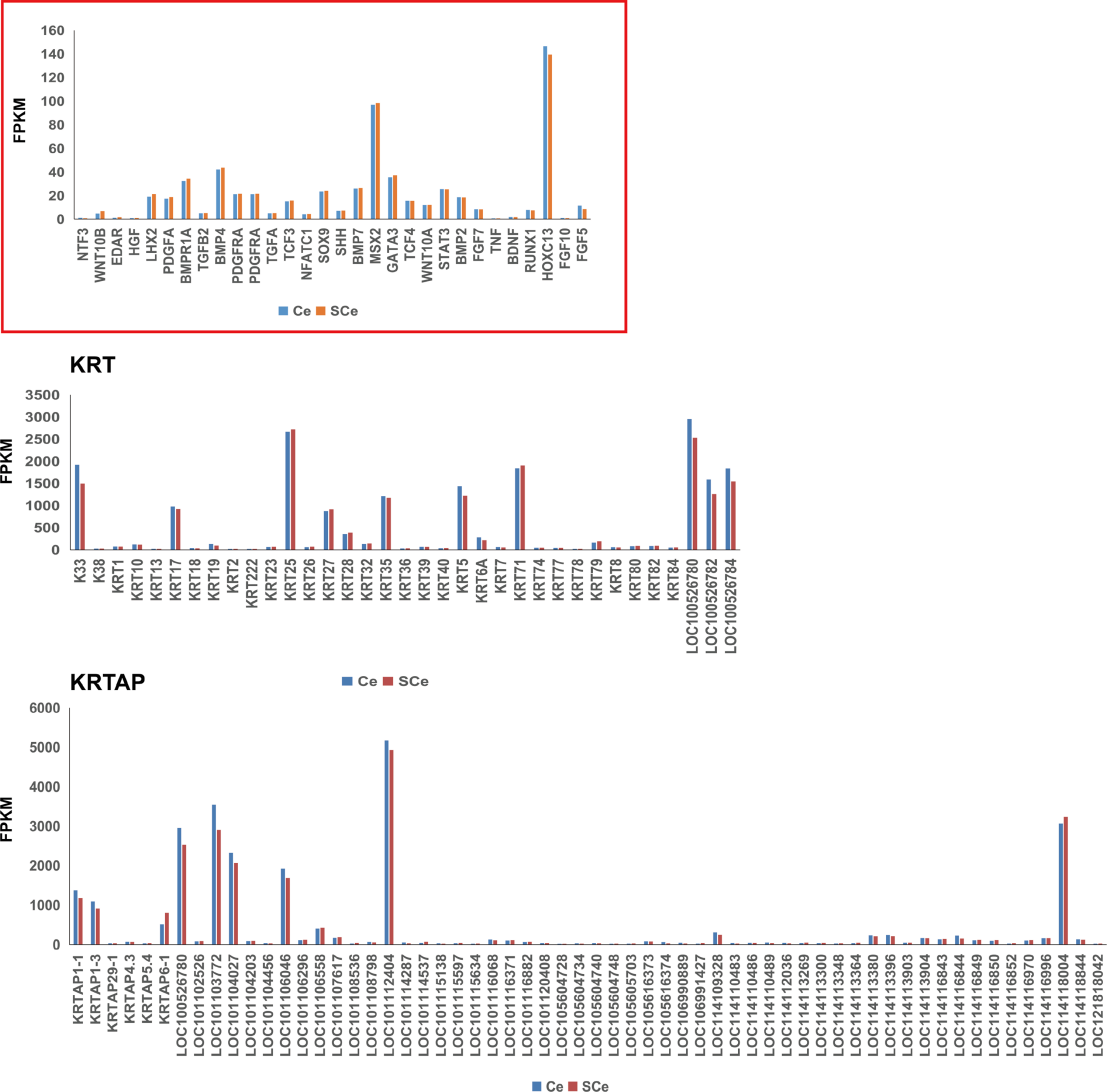
The expression patterns of genes related to growth and development of hair follicle/hair, *KRT* and *KRTAP* gene family members in skin tissues of Subo and Chinese Merinos.

For a long time, molecular signaling pathways and candidate genes that affect the development, cycle and hair growth of animal hair follicles have been the focus of attention (Table 1). Among them, the well-known pathways are BMP (Bone morphogenetic protein), MAPK (Mitogen activated protein kinase), Wnt, Notch and Sonic Hedgehog (Shh) signaling pathways. A large number of previous studies have confirmed that some classical genes are involved in these pathways, for example, *BMP4*, *BMP7*, *Wnt5*, *Wnt10* and *SHH* genes, etc. We summarized these genes in detail, and paid attention to the expression of them in the skin tissues of adult Chinese and Subo Merinos. The results showed that, the expression of *HOXC13* and *MSX2* genes in skin tissues was the highest among all the counted genes, which may be related to their strong wool growth at this time (Fig. 2). In addition, the expression levels of genes (*e.g.*, *LHX2*, *PDGFA* and *Wnt10A* genes, etc.) associated with wool follicle morphogenesis or cycle were significantly lower than those of the above two genes, which may be due to the vigorous growth and maturity of hair follicles in embryos rather than in adulthood (Fig. 2).

**Table 1:** The candidate genes that have been reported for growth and development of hair follicle or hair.

**Genes**	**Functions**	**References**
***LHX2, NFATC1, RUNX1, SOX9, STAT3, TCF3*** **and** **TCF4**	Affecting stem cell quiescence and activation, and subsequent hair regrowth during the hair cycle	Nowak et al. (2008), Rhee, Polak & Fuchs (2006), Vidal et al. (2005), Horsley et al. (2008), Nguyen, Rendl & Fuchs (2006), Nguyen et al. (2009), Sano et al. (2000) and Osorio et al. (2008)
***WNT10B, EDAR, DKK4 and K17***	Patterning the epidermis before any visible signs of hair placodes	Reddy et al. (2001), Headon & Overbeek (1999), Bazzi et al. (2007) and McGowan & Coulombe (1998)
***SHH, PDGFA, FGF*** **and** **TGFB2**	Promoting hair germ formation	Sennett & Rendl (2012)
***WNT10A and WNT10B***	Perpetuating focused Wnt signaling activity within both placode and condensate in the placode as morphogenesis begins	Reddy et al. (2001)
***DKK4, BMP4*** **and** **BMP7**	Suppressing placodeinduction	Sick et al. (2006) and Pummila et al. (2007)
***BMPR1A*** **and** **BMP2**	Affecting early follicle formation	Bitgood & Mcmahon (1995)
***PDGF*** **and** **PDGFRA**	The maintenance of the dermal papilla	Karlsson, Bondjers & Betsholtz (1999)
***FGF7*** **and** **FGF10**	Organizing hair growth during postnatal morphogenesis	Guo, Degenstein & Fuchs (1996)
***EGF, IGF, TGFA, CUTL1, GATA3, HOXC13, FOXN1*** **and** **MSX2**	Affecting hair shaft differentiation, structure and shape	Schlake (2007)
***FGF5***	Ppromoting catagen entry	Hébert et al. (1994)
***BDNF, IL1B, NTF3, TGFB1*** **and** **TNF**	Advancing anagen/catagen transition	Stenn & Paus (2001) and Paus & Foitzik (2004)
***HGF, IGF1 and VEGF***	Promoting anagen maintenance	

In addition, keratin proteins (KRTs) and keratin-associated proteins (KRTAPs) are the structural protein of animal hair/wool. The *KRT* and *KRTAP* gene families consist of many genes with similar structures and functions. It is the first-choice candidate gene when looking for major genes controlling hair/wool traits. Among these genes, we found that *K33*, *KRT17*, *KRT25*, *KRT27*, *KRT35*, *KRT5*, *KRT71*, *LOC100526780*, *LOC100526782*, *LOC100526784*, *KRTAP1-1*, *KRTAP1-3*, *LOC100526780*, *LOC101103772*, *LOC101104027*, *LOC101106046*, *LOC101112404*, *LOC114118004* are expressed at relatively higher levels in skin tissues (Fig. 2). Among these 18 genes, the expression of most genes in skin tissues of Chinese Merinos is slightly higher than that of Subo Merinos (Fig. 2).

### Differential expression analysis

**Figure 3:**
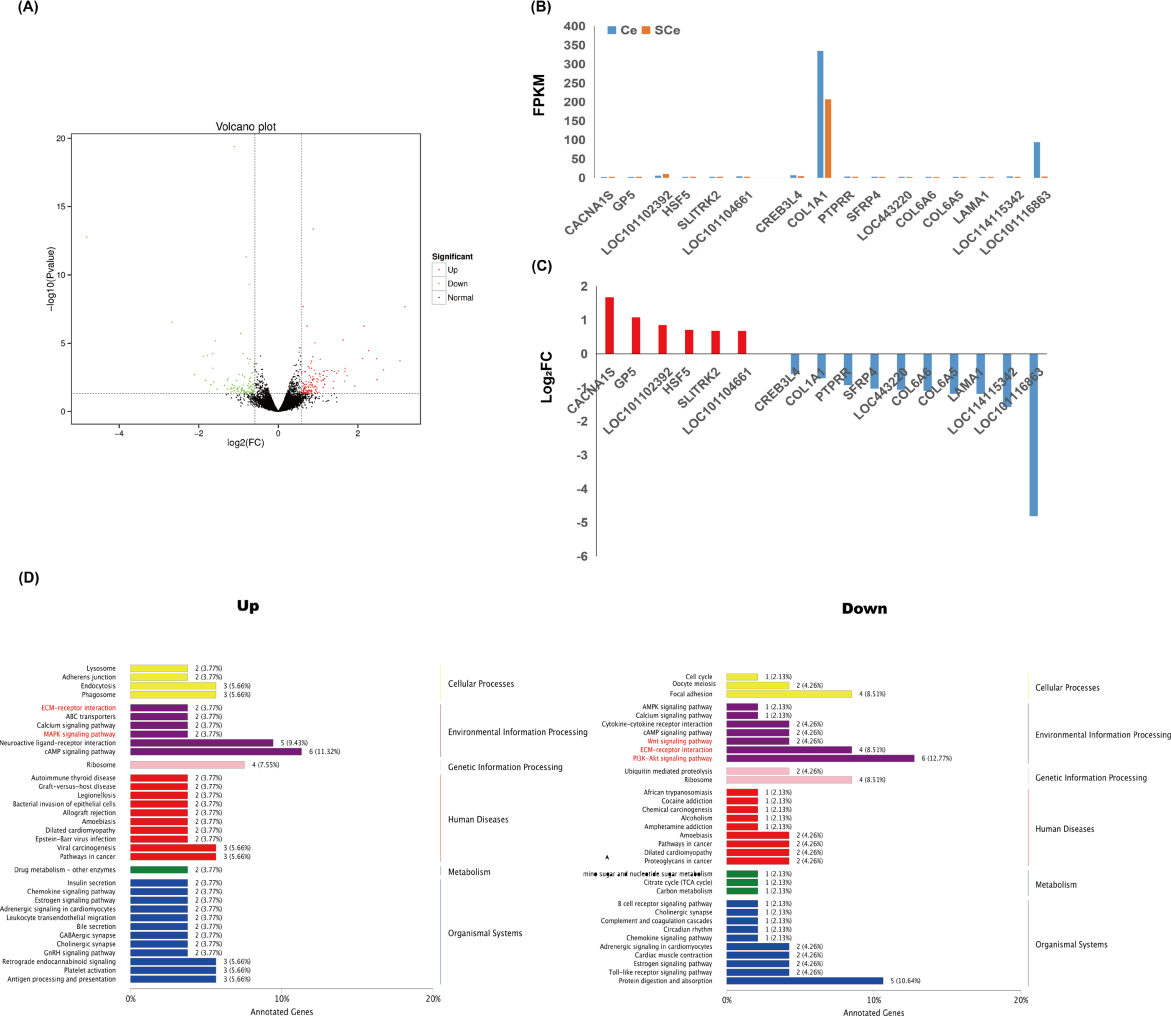
Transcriptome analysis of skin tissues of Subo and Chinese Merinos. (A) Volcano plot of DEGs. (B) The Expression patterns of 16 DEGs in skin tissues of Subo and Chinese Merinos. (C) The Log_2_FC of 16 DEGs. (D) KEGG enrichment analysis of up-regulated and down-regulated DEGs.

The skin transcriptomes of four Subo Merinos were used as the experimental group and four Chinese Merinos were used as the control group. The experimental group was compared with the control group. The analysis results are shown in the figure. In the figure, green points represent down-regulated differentially expressed genes, red points represent up-regulated differentially expressed genes, and black points represent non-differentially expressed genes. A total of 236 differentially expressed genes were screened in SCe *vs.* Ce, among which 139 were up-regulated and 97 were down-regulated (Fig. 3A).

### GO enrichment of DEGs

GO functional enrichment analysis was performed on the up-regulated DEGs and the down-regulated DEGs, respectively. The results showed that the up-regulated DEGs were mainly enriched in negative regulation of cell differentiation (GO:0045596), exocytosis (GO:0006887), postsynaptic density of dendrite (GO:0014069), glutamatergic synapse (GO:0098978), etc (Fig. S1). The down-regulated DEGs were mainly enriched in extracellular region (GO:0005576), extracellular space (GO:0005615), extracellular matrix structural constituent (GO:0005201) and calcium ion binding (GO:0005509), *etc*. (Fig. S1).

### KEGG enrichment of DEGs

**Figure 4:**
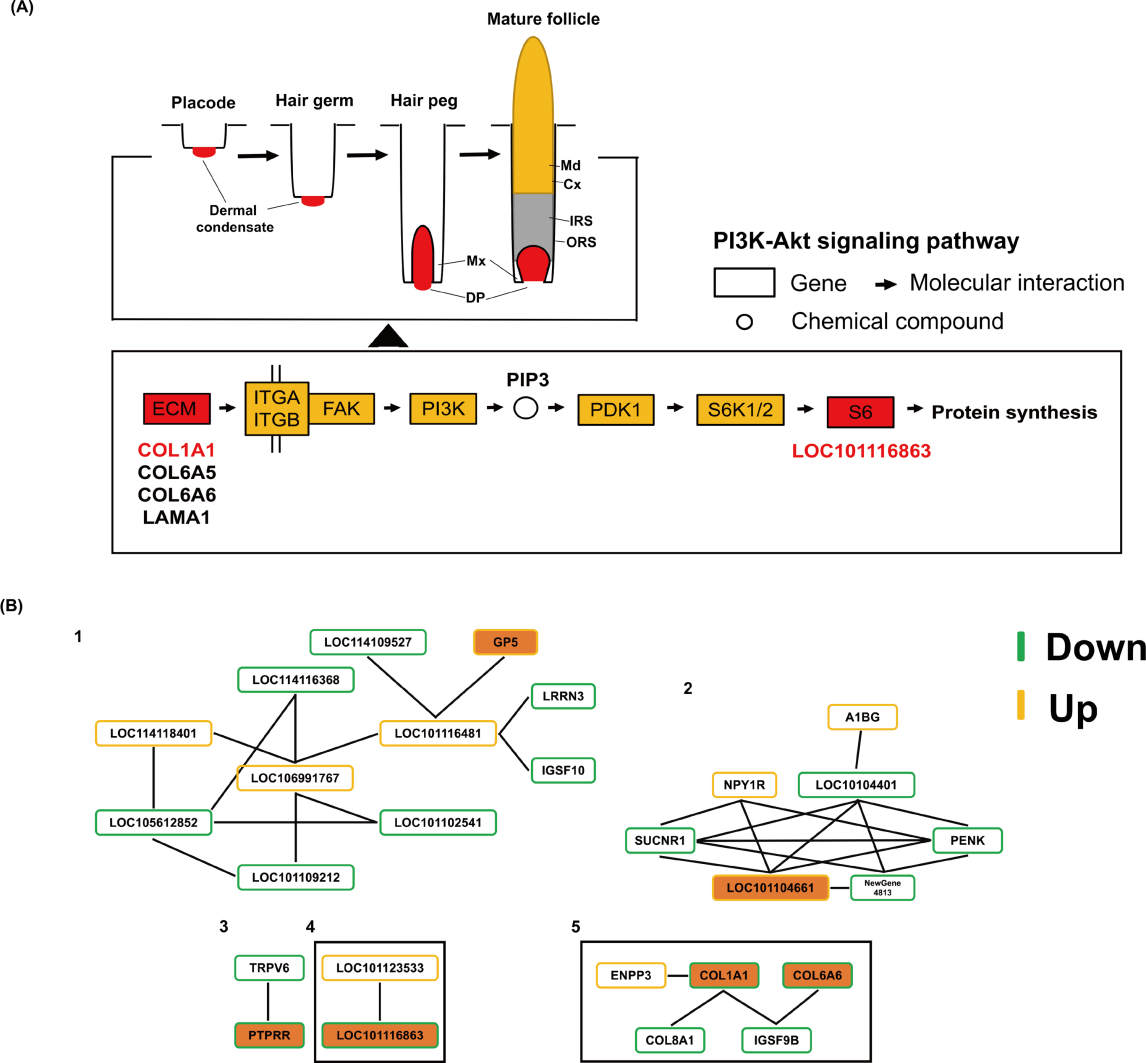
The potential regulatory roles of important DEGs on hair/wool follicle growth and development. (A) The positions of 5 DEGs in the PI3K-Akt signaling pathway. (B) Protein protein interaction networks of DEGs.

KEGG functional enrichment was performed on up-regulated and down-regulated DEGs respectively, and the enriched pathways were mainly divided into six categories: Cellular Processes, Environmental Information Processing, Genetic Information Processing, human diseases, Metabolism and Organic Systems (Fig. 3D). It is worth noting that in the up-regulated part, *GP5*, *SLITRK2* genes and *CACNA1S*, *LOC101102392* genes were enriched in ECM-receptor interaction and MAPK signaling pathway, respectively, and *HSF5* gene was enriched in Wnt signaling pathway. In the down-regulated part, the *CREB3L4* gene was enriched in the AMPK signaling pathway. In addition, two (*LOC114115342* and *SFRP4* genes), four (*COL1A1*, *COL6A5*, *COL6A6* and *LAMA1* genes), six (*COL1A1*, *COL6A5*, *COL6A6*, *CREB3L4*, *LAMA1* and *LOC101116863* genes) DEGs were enriched for Wnt signaling pathway, ECM-receptor interaction and PI3K-Akt signaling pathway (Fig. 4A).

### Protein protein interaction network of DEGs

In order to further understand the relationship between DEGs, we constructed the network interaction network of DEGs. The results showed that a total of 27 differentially expressed genes formed five protein protein interaction networks (Fig. 4B). Among them, the interaction relationship in the second protein network interaction is the most complex. The DEG *LOC101104661* is a core node with a node degree of five, which is enriched in the TNF signaling pathway. Notably, *GP5*, *LOC101104661*, *PTPRR*, *LOC101116863*, *COL1A1* and *COL6A6* genes were included in these five protein interaction networks, respectively. Among these genes, there was an interaction relationship between the differentially expressed genes *LOC101116863* and *LOC101123533*, and *LOC101123533* was enriched in the Ribosome pathway, which also suggested that LOC101116863 was related to protein synthesis. In addition, in the last protein interaction network, the differentially expressed gene *COL1A1* is the core node, the node degree is three, and there is an interaction relationship between *COL8A1* and *COL1A1* genes.

### Nucleic acid/protein sequence structure and phylogenetic analysis of *COL1A1* and *LOC101116863* genes

**Figure 5:**
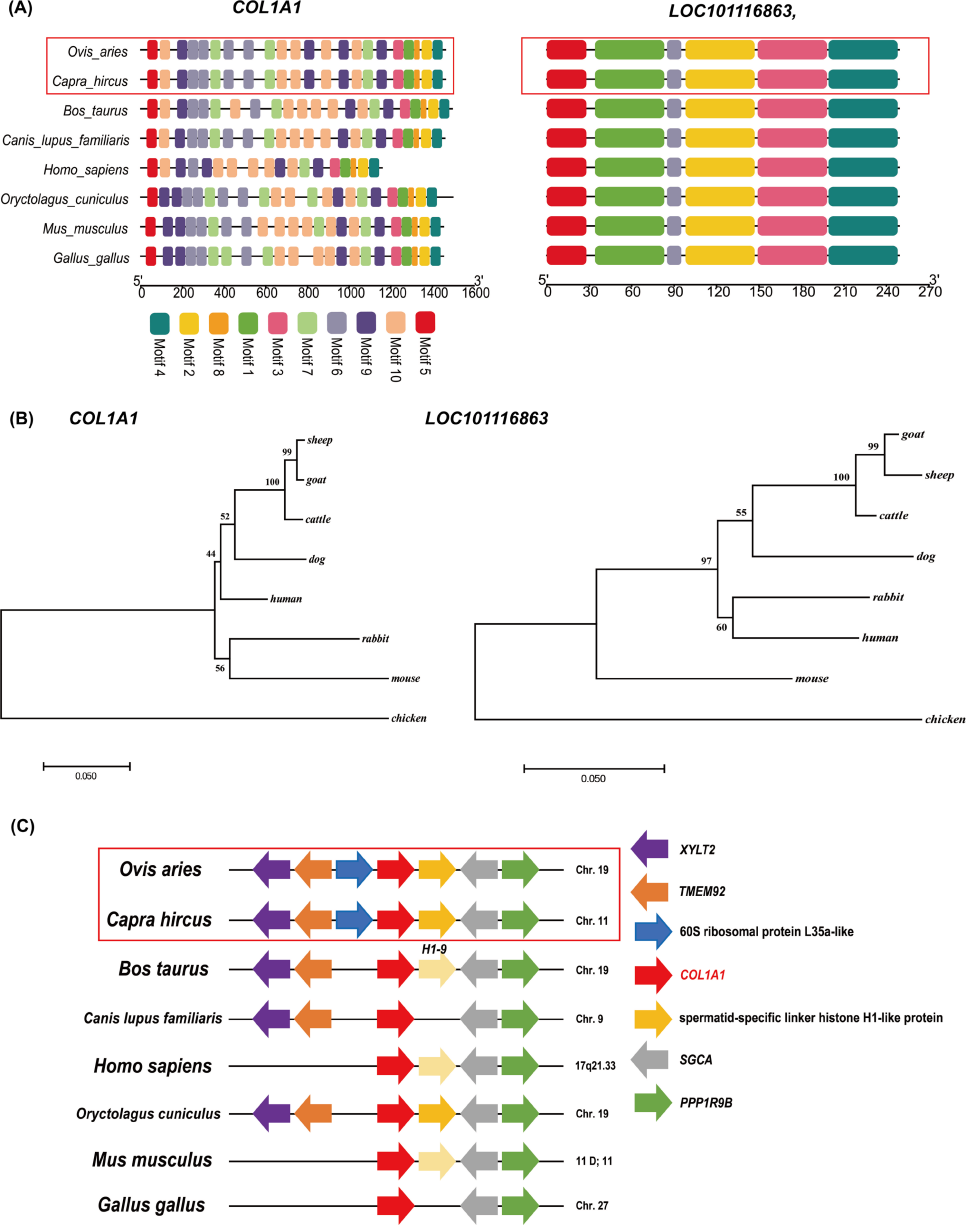
Bioinformatics analysis of *COL1A1* and *LOC101116863* genes. (A) The prediction of protein sequence domains of COL1A1 and LOC101116863 in eight species. (B) Phylogenetic analysis of *COL1A1* and *LOC101116863* genes. (C) Synteny analysis of *COL1A1* genes in eight species. The direction of the arrow in the figure represents the transcription direction of the gene.

Next, we focused on DEGs that were enriched in ECM-receptor interaction, MAPK signaling pathway, Wnt signaling pathway, AMPK signaling pathway, and the PI3K-Akt signaling pathway. Among these DEGs, the expression level of *COL1A1* gene is the highest in the skin tissues of Subo or Chinese Merinos (Figs. 3B and 3C). At the same time, the fold change of *LOC101116863* gene is the largest. Notably, these two genes are located at the head and tail of a coherent signaling pathway regulating protein biosynthesis (ECM-PI3K). We further compared the nucleic acid and protein sequence structures of *COL1A1* and *LOC101116863* genes among the eight species (Files S3 and S4), and the results showed the high nucleic acid sequence similarity of *COL1A1* or *LOC101116863* genes in the eight species. In addition, the COL1A1 or LOC101116863 proteins of the eight species all have multiple common domains (Fig. 5A). Compared with other animals, the human COL1A1 protein appears to be missing several common domains, with COL1A1 proteins having the highest domain similarity in goat and sheep. Meanwhile, the LOC101116863 protein showed a more conserved structure in different species. Phylogenetic analysis further showed the high homology of bovine, goat and ovine *COL1A1* genes, and *LOC101116863* gene in sheep was relatively more primitive (Fig. 5B). Synteny analysis further showed that the *COL1A1* gene has more than 3 fixed neighbors on the chromosome in eight species. Meanwhile, sheep *COL1A1* and goat *COL1A1* both have six fixed neighbors (Fig. 5C). This suggseted that the chromosomal segment where the *COL1A1* gene is located, is very conserved in sheep and goats, and is some what conserved in other six species. However, no similar features were found in the *LOC101116863* gene.

### Selection pressure analysis of *COL1A1* and *LOC101116863* genes

In the selection pressure analysis, M0, M1, M2, M7 and M8 models were used to analyze the selection pressure of *COL1A1* and *LOC101116863* genes during the species evolution, respectively. M0 model analysis results showed that *ω* values of *COL1A1* and *LOC101116863* genes were all less than 1, indicating that the they are affected by negative selection (Table 2). M1 model analysis results also showed that the *ω* values of them were ≤1 (Table 2). In addition, M2 model analysis found that the mRNA sequences of *COL1A1* gene contained 40 positive selection loci; M8 model analysis found that the mRNA sequences of *COL1A1* gene contained 51 positive selection loci (Table 2). These results suggested that *COL1A1* and *LOC101116863* genes were affected by negative/purify selection. This further indicates that the functions of the two genes may be conserved to a certain extent.

**Table 2:** Selection pressure analysis of COL1A1 and LOC101116863 genes.

**Gene**	**Name**	**Model code**	**lnL**	**Parameters**	**Number of positive selection sites**
**COL1A1**	M0(one-ratio)	M0(one-ratio)	−13632.11088	*w* = 0.09073	none
	M1(Nearly Neutral)	M1(Nearly Neutral)	−13420.78598	p:0.89963 0.10037 w:0.02464 1.00000	Not allowed
	M2(positive Selection)	M2(positive Selection)	−13420.78598	p:0.89963 0.06374 0.03662 w:0.02463 1.00000 1.00000	40
	M7(beta)	M7(beta)	−13419.66591	*p* = 0.06938*q* = 0.54218	Not allowed
	M8(beta&w >1)	M8(beta&w >1)	−13415.39886	*p*0 = 0.98812*p* = 0.08993*q* = 0.86629 (*p*1 = 0.01188) *w* = 2.82510	51
**LOC101116863**	M0(one-ratio)	M0(one-ratio)	−2155.510075	*w* = 0.01231	none
	M1(Nearly Neutral)	M1(Nearly Neutral)	−2155.512246	p:0.99999 0.00001 w:0.01231 1.00000	Not allowed
	M2(positive Selection)	M2(positive Selection)	−2155.510075	p:1.00000 0.00000 0.00000 w:0.01231 1.00000 58.25826	none
	M7(beta)	M7(beta)	−2155.71926	*p* = 1.29084*q* = 99.00000	Not allowed
	M8(beta&w >1)	M8(beta&w >1)	−2155.721426	*p*0 = 0.99999*p* = 1.29082*q* = 99.00000(*p*1 = 0.00001) *w* = 1.00000	none

## Discussion

Wool fineness is an important factor in determining the economic value of wool. Genetic improvement of wool fineness traits has been carried out in a wide range of sheep breeds. However, this work has been slow for a long time. In recent years, with the rise of molecular breeding technology, studying the molecular mechanism regulating the wool fiber fineness has become a focus of attention. Screening candidate genes associated with wool fineness by RNA-Seq provide new ideas for us to study this molecular mechanism, and theoretical references for MAS or GS of sheep breeds.

It is widely known that the hair/wool follicle is the control center of hair/wool growth and development, and further determines the fineness of the wool in different sheep breeds. Numerous studies have shown that, many molecular signaling pathways regulate the hair follicle growth and development. For example, Andl et al. (2002) found that Wnt signaling pathway is required for the initiation of hair follicle growth. Sohn et al. (2015) found that the promotion effects of hair growth are mediated by the activation of Wnt/β-catenin and MAPK signaling pathways. Lu et al. (2021) found that amphiregulin promotes hair regeneration of skin-derived precursors *via* the PI3K and MAPK signaling pathways. Based on the identification of DEGs, we found 16 DEGs (Included: *CACNA1S*, *GP5*, *LOC101102392*, *HSF5*, *SLITRK2*, *LOC101104661*, *CREB3L4*, *COL1A1*, *PTPRR*, *SFRP4*, *LOC443220*, *COL6A6*, *COL6A5*, *LAMA1*, *LOC114115342* and *LOC101116863* genes) located in the above pathways, these genes can be used as primary candidate genes for regulating the wool fineness in Merinos. In addition, a few reports, *e.g.*, Zhao et al. (2017) compared the transcriptomes of skin tissues from backs and bellies of Chinchilla rex rabbits (*Oryctolagus cuniculus*), and found that *CACNA1S* and *SFRP2* genes associated with Hair follicle and skin development. Yue et al. (2016) found that *COL6A6* gene associated with hair follicle morphogenesis in sheep by using strand-specific RNA sequencing. Nie et al. (2018) found that *LAMA1* gene mainly influence epidermal and wool placode development in carpet sheep fetal skin based on RNA-Seq. These findings also support our conclusions.

It is worth noting that, among these 16 DEGs, the fold change value of *LOC101116863* gene is the largest. Meanwhile, the expression of *COL1A1* gene in Merino skin tissue was significantly higher than that of the other 16 DEGs (the expression levels of these 15 DEGs in skin tissue were extremely low), suggesting that *COL1A1* gene may play important roles in skin tissue of Merinos. Further based on multiple sequence alignment and synteny analysis, it was found that the protein sequence structures of *COL1A1* and *LOC101116863* genes are very conserved in eight species (among which the structures of sheep *COL1A1* gene or *LOC101116863* gene are very similar to that of goats, respectively). Phylogenetic analysis showed that, the *COL1A1* and *LOC101116863* gene sequences of bovine, goat and sheep are highly homologous, respectively. The selection pressure analysis showed that both *COL1A1* and *LOC101116863* genes were affected by negative selection. These results final suggest that, *COL1A1* and *LOC101116863* genes may have similar functions in goats, sheep and cattle respectively, and their functions are partly conserved. In a past studies, Cai et al. (2022) revealed the SNPs of *COL1A1* gene with cashmere production performanc in Liaoning cashmere goats based on association analysis. Wang et al. (2021) also suggested that, *COL1A1* gene as a key candidate gene for regulating cashmere production in goat by transcriptome analysis. Zhang et al. (2021) also found that *COL1A1* and *COL65A* genes were associated with cashmere fineness by transcriptome analysis. These results all emphasize that *COL1A1* gene regulates cashmere yield and fineness in goats. Therefore, we consider that *COL1A1* and *LOC101116863* genes are the most important among the 16 candidate genes associate with wool fineness, but the specific regulatory roles of these genes on wool fineness still need to be further verified. In addition, combined with the results of protein domain prediction, we also found that the structure of human COL1A1 protein is quite different from other animals (included: sheep, goat, cattle, dog, rabbit and chicken). Hence, we also speculate that the structural differences of COL1A1 protein may be the reason for the obvious difference in hair distribution and amount of hair between humans and other animals.

## Conclusion

Based on the comparison between the skin transcriptomes of Subo and Chinese Merinos, a total of 16 candidate DEGs associated with wool fineness (Included: *CACNA1S*, *GP5*, *LOC101102392*, *HSF5*, *SLITRK2*, *LOC101104661*, *CREB3L4*, *COL1A1*, *PTPRR*, *SFRP4*, *LOC443220*, *COL6A6*, *COL6A5*, *LAMA1*, *LOC114115342* and *LOC101116863* genes) were screened, which were enriched in signal pathways related to the regulation of hair or hair follicle growth. Among these genes, *COL1A1* and *LOC101116863* genes may be the most critical, and the conserved features of their sequence structures may make them have similar regulatory functions in different species.

##  Supplemental Information

10.7717/peerj.15327/supp-1 Supplemental Information 1 Figure S1 GO enrichment analysis of DEGs

10.7717/peerj.15327/supp-2 Supplemental Information 2 Table S1 The wool spinning numbers of 8 Merinos and data quality control of 8 Merino transcriptomes

10.7717/peerj.15327/supp-3 Supplemental Information 3 Table S2 The log2FC of all genes

10.7717/peerj.15327/supp-4 Supplemental Information 4 COL1A1.fas: The multiple sequence alignment of *COL1A1* genes of eight species

10.7717/peerj.15327/supp-5 Supplemental Information 5 LOC101116863.fas: The multiple sequence alignment of *LOC101116863* genes of eight species

10.7717/peerj.15327/supp-6 Supplemental Information 6 Raw Data

10.7717/peerj.15327/supp-7 Supplemental Information 7 ARRIVE 2.0 Checklist

## Additional Information and Declarations

### Data Availability

The following information was supplied regarding data availability: The eight skin transcriptomes’ data are available at NCBI’s SRA: PRJNA889222.
